# Yessotoxin, a Promising Therapeutic Tool

**DOI:** 10.3390/md14020030

**Published:** 2016-01-28

**Authors:** Amparo Alfonso, Mercedes R. Vieytes, Luis M. Botana

**Affiliations:** 1 Department of Pharmacology, Faculty of Veterinary, University of Santiago of Compostela, 27002 Lugo, Spain; 2 Department of Physiology, Faculty of Veterinary, University of Santiago of Compostela, 27002 Lugo, Spain; mmercedes.rodriguez@usc.es

**Keywords:** yessotoxin (YTX), apoptosis, autophagy, cellular death, signal transduction, cytoskeleton, immune system, Alzheimer, glucose metabolism

## Abstract

Yessotoxin (YTX) is a polyether compound produced by dinoflagellates and accumulated in filter feeding shellfish. No records about human intoxications induced by this compound have been published, however it is considered a toxin. Modifications in second messenger levels, protein levels, immune cells, cytoskeleton or activation of different cellular death types have been published as consequence of YTX exposure. This review summarizes the main intracellular pathways modulated by YTX and their pharmacological and therapeutic implications.

## 1. Introduction

Phycotoxins are secondary metabolites produced by dinoflagellates and diatoms. Under this term, several compounds with different chemical properties, structures and mechanisms of action are included. Of these, yessotoxin (YTX) and its analogues, YTXs, are one of the most interesting groups for different reasons. YTX is an exotoxin, released by producing cells [1]. Its ecological role is unknown, and its inclusion in the list of marine toxins is due to the fact that it coexists with diarrheic toxins (okadaic acid and dinophysistoxins) and causes mice death after intraperitoneal injection, although low oral toxicity has been reported and no records about human intoxications have been reported [2,3]. The European Food Safety Authority (EFSA) working group on marine toxins proposed an acute reference dose (ARfD) of 25 µg YTX equivalents/kg body weight [4]. The European Union (EU) has recently elevated the toxin limit from 1 to 3.75 mg of YTX equivalent/kg of shellfish meat, while the limit for the other toxins within the lipophilic group is 160 µg of toxin equivalent/kg of shellfish meat [5]. This is a preventive measure, since, as mentioned, intoxications in humans with YTXs have never been reported. In addition, YTX is poorly absorbed after oral administration and most of the toxin is recovered from the lower intestine and feces [3]. Besides, only ultrastructural cardiac damages without other alterations were reported after oral, intraperitoneal or intravenous administrations to rats and mice [6,7,8]. The same effects were reported in mice after oral co-administration of YTX and okadaic acid [9] and no toxic effects were observed after oral exposure when YTX was combined with azaspiracid-1 [10]. Controversial data were published after intraperitoneal administration. In these senses, a wide range of lethal doses (LD_50_) have been reported, from 80 to 750 µg/kg, with erratic and no well-defined tissue alterations [2,8,11,12,13,14,15]. Therefore, YTX is a different “toxin”.

## 2. YTX Origin

YTX was first isolated in 1986 in Mutsu Bay, Japan from digestive glands of scallops *Patinopecten yessoensis* after a food intoxication episode [16]. Later, *Protoceratium reticulatum*, *Lingulodinium polyedrum* and *Gonyaulax spinifera* were identified as the dinoflagellates that produce this toxin [16,17,18,19]. In addition to Japan, YTX has been identified in shellfish harvested in Europe, including Spain, Italy, Norway, the Adriatic Sea, and Russia; Chile; and New Zealand [20,21]. The toxin has been mainly localized in immunocytes and in the digestive gland of mussels [22].

## 3. YTX Chemistry

**Figure 1:**
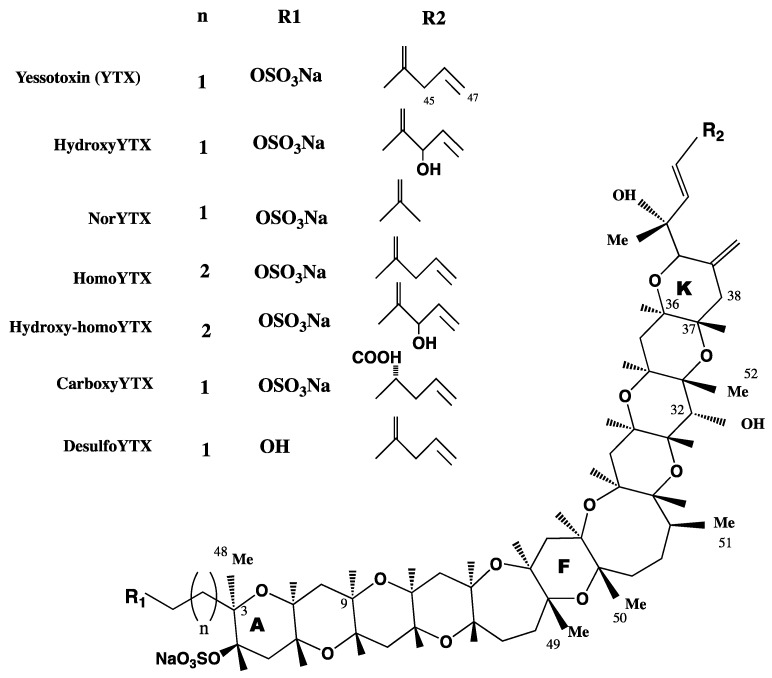
Structure of yessotoxins (YTXs).

YTXs are a group of ladder-shaped polycyclic and polyether compounds (Figure 1). YTX planar structure was first described in 1987 and the absolute configuration was reported in 1996 [16,23,24]. More than 90 analogues have been so far described, although not all have been structurally identified and isolated [25]. The chemical structure of YTXs, 11 adjacent ether rings, resembles those of other phycotoxins, such as brevetoxins, ciguatoxins or gambierone [26]. However, the cellular target of these toxins, the sodium channel, and the one for YTX are totally different. In addition, YTXs structure has an unsaturated side chain of nine carbons (C9), with different functional groups and one or more sulfate, which increase the polarity of the molecule [27]. Some YTXs are directly produced by dinoflagellates, while others are produced after shellfish metabolism. In this sense, YTX and homo YTX are produced by dinoflagellates, while 45-OH-YTX or carboxy-YTX were only isolated from shellfish [17,28,29]. In these animals, YTXs suffer extensive metabolism with a half-life of 20–24 days [30]. YTXs producing dinoflagellates coexist with those producing okadaic acid, and for this reason YTXs were considered diarrheic toxins for some years. Now, their toxicological and chemical characteristics, as well as the biogenetic origins, are better known and since it has been demonstrated that YTXs do not produce diarrhea, these toxins are considered in different groups [31,32].

## 4. Mechanism of Action of YTX

To clarify the mechanism of action of YTXs, the modulation of several second messengers and proteins have been studied. Since YTX was considered a diarrheic toxin, first the effect on serine/threonine protein phosphatases (PP) PP1 and PP2A was checked. YTX was four times less effective than okadaic acid to inhibit these enzymes [33]. Therefore, it was concluded that YTX effect was not mediated by these phosphatases.

Depending on the cellular model, different effects in cytosolic calcium and adenosine 3′,5′-cyclic monophosphate (cAMP) levels have been reported after YTX incubation. A fast, but small, increase in cytosolic calcium levels in human fresh lymphocytes was observed. This increase was due to the activation of nifedipine and SKF 96365 sensitive calcium channels. In this cellular model, the influx of calcium through the store operated calcium channels was inhibited by YTX [34]. However, in the tumor model erythroleukemia K-562 cell line, YTX did not directly modify cytosolic calcium levels, but it increased thapsigargin-independent calcium pools depletion, as well as the influx through store operated calcium channels [35]. A cytosolic calcium increase in the presence of YTX was also observed in HL7702 human liver cells, Bel7402 human hepatoma cell line and in primary cultures of rat cerebellar neurons [36,37,38,39]. However, no direct modification in calcium basal levels was observed in rat primary cardiomyocytes after YTX incubation [40]. The effect over calcium in the K-562 cell line was related with the cAMP pathway and with the mitochondria activity [35]. In addition, permissive levels of calcium were necessary to observe the effect of YTX over the mitochondrial membrane in hepatic cells [41]. In this sense, these organelles were also affected in cardiomyocytes and neuroblastoma cells after YTX exposure [11,42]. Besides, changes in permeability of the outer mitochondrial membrane and production of pro-apoptotic factors together with swelling of mitochondria were described after incubation with YTX in myoblast cell lines [43]. Also, changes in mitochondrial membrane potential and the opening of the permeability transition pore were described after YTX exposure of hepatic cells [41].

Opposite effects were also observed in cAMP levels. In the presence of calcium, YTX dose-dependent decreased cAMP levels in human fresh lymphocytes, while a significant increase was observed in the same conditions in the K-562 cell line. These effects were calcium-dependent and also observed in guanine 3′-5′cyclic monophosphate (cGMP) levels [35,44,45,46]. On the other hand, YTX did not directly modify cAMP levels in rat primary cardiomyocytes, although the toxin significantly decreased the levels of this second messenger when the synthesis was activated [40].

The levels of cyclic nucleotides are regulated by phosphodiesterases (PDEs). These are a group of isozyme families with different substrate specificity, affinity, tissue localization and sensitivity to inhibitors. YTX was described to activate PDEs in human T lymphocytes in parallel with the decrease in cAMP levels. This effect was calcium dependent and modulated by PDEs activators and inhibitors [44]. The binding of YTX to PDEs was later showed within a resonant mirror biosensor where the kinetic equilibrium dissociation constant (K_D_) for the binding of YTXs and different PDEs was studied. In this way, K_D_ (3.74 ± 0.08 µM YTX) and the structure–selectivity relation of YTX–PDEs association was described [47,48,49,50]. In addition, the binding was confirmed by measuring changes in fluorescence polarization of a PDE-dye conjugate in the presence of YTX [51]. Thus, it was concluded that YTXs bind to PDE1, PDE3 and PDE4 and to exonuclease PDE I.

From all this information a relation between YTX, PDEs, calcium levels and mitochondrial conditions was concluded. The functional link between PDEs and mitochondria are the A-kinase anchor proteins (AKAPs). These are a big group of proteins that integrate PDEs and the protein kinase A (PKA), which is the kinase activated by cAMP. The cellular localization of AKAP-PDE-PKA complex is a key step in cAMP compartimentalisation signaling [52,53]. There are several AKAPs families, neuronal AKAPs such as AKAP 79, organelle-associated AKAPs such as AKAP 149 associated to the mitochondria, gravin AKAPs (AKAP 250) associated to membrane receptors and AKAP 95 associated to the nuclear matrix [54]. After YTX incubation, a significantly decrease of AKAP149 cytosolic levels in the erythroleukemia K-562 cell line was observed, which led to cell death. On the contrary, in fresh human lymphocytes, cytosolic AKAP149 levels were significantly increased after toxin incubation and cells survived [35]. The meaning of these results was further studied and a key role of cellular distribution of AKAP-149-PKA-PDE4A complex in YTX effect was reported. After 24 h of YTX exposure, the AKAP 149-PKA-PDE4A complex was located in the plasma membrane and apoptosis was activated, while after 48 h of YTX treatment the complex was located in the nuclear domain and non-apoptotic cellular death was observed. None of these effects were shown when AKAP-149 or PDE4A were silenced [55].

Protein kinase C (PKC) is a protein involved in multiple and different biological events and thus it plays a crucial role in cell metabolism regulation. Besides, PKC are a family of enzymes involved in pro-survival or pro-apoptotic events. Several subtypes of PKC were reported to affect some metabolic pathways activated by YTX in mice primary cortical neurons and in the mouse T lymphocyte cell line EL-4 [56,57]. PKCs modulation induced by YTX in the K-562 cell line was PDE4A-dependent, since the silencing of this protein changed PKC expression pattern [58]. However, the effect of YTX described over cortical neurons with triple transgenic mutation for Alzheimer’s disease (3× Tg-AD) mediated by PKC was not related with PDEs or PKA [56]. This difference could be due to the lack of drug specificity or to the cellular model used.

**Figure 2:**
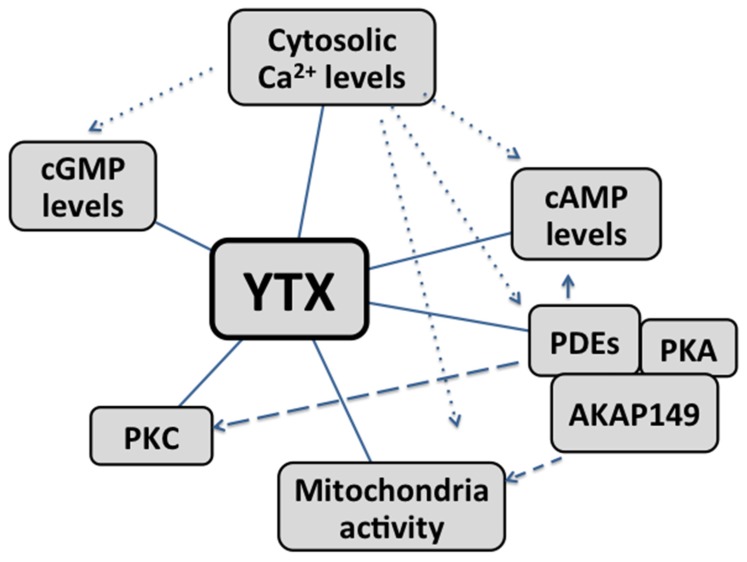
Cross-talks between second messengers and main intracellular organelles involved in the mechanism of action of YTX. Straight lines: pathway directly modulated by YTX. Dotted lines: Calcium levels affect YTX modulation on cGMP, cAMP, AKAP149-PKA-PDEs complex and mitochondria activity. Dashed lines: the effect of YTX is possibly mediated by the modulation of AKAP149-PKA-PDEs complex.

In summary, cAMP, calcium, PDEs, PKC and AKAP-149 as well as mitochondria, are involved in the mechanism of action of YTX (Figure 2). The role of each and the final effect depend on the cellular model studied.

## 5. Cellular Death and YTX

**Figure 3:**
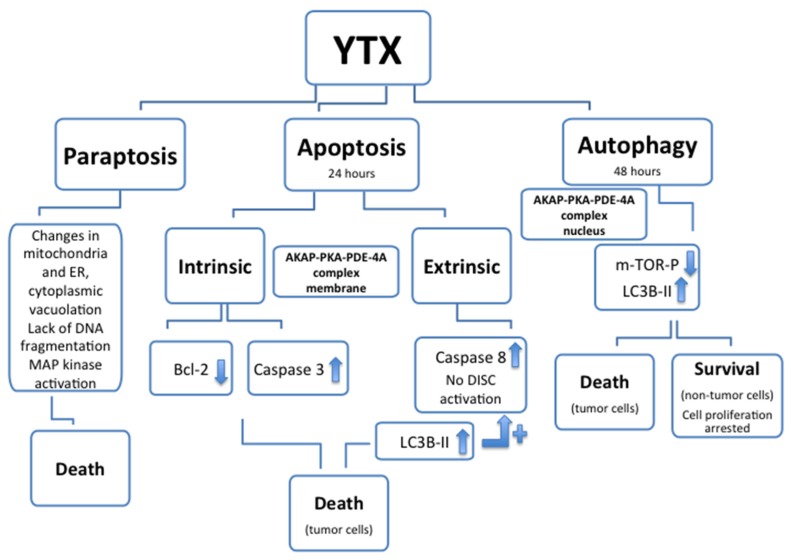
Cellular death types (paraptosis, apoptosis and autophagy) and associated hallmarks affected after YTX treatment. In some cellular models, after YTX treatment, autophagy is activated and cellular proliferation arrested, however no cellular death is associated.

Apoptotic and non-apoptotic cellular death were above mentioned as consequence of YTX exposure. The apoptotic effect of YTX had been reported for the first time in 2002 in BE(2)-M17 neuroblastoma cell line [42]. Later, several apoptosis hallmarks in many different cell types, such as HeLa S_3_ cells, cerebellar neurons, L6 and BC3H1 myoblasts, mouse fibroblasts NIH3T3, MDCK kidney cells and MCF-7 breast cells, HepG2, Bel7402 and HL7702 human hepatoma cell lines, and liver cells have been published [37,38,39,43,59,60,61,62,63,64]. In the erythroleukemia K-562 cell line the apoptotic cell death was activated after 24 h YTX incubation when the AKAP 149-PKA-PDE4A complex was located in the plasma membrane [55]. In these cells, variations of both intrinsic (Bcl2 and caspases 3) and extrinsic (caspase 8) apoptotic hallmarks had been showed after 24 h of YTX incubation, indicating the activation of these two types of apoptotic cell death [55,65]. After YTX exposure, no external ligands to activate death-inducing signaling complex (DISC) were described [66]. Therefore the increase of caspase 8 activity, and as consequence the activation of extrinsic apoptosis, may be mediated by the activation of internal receptors (Fas receptor) through the light chain-3B-II (LC3B-II) protein increased after 24 h of YTX exposure [67]. The non-apoptotic programed cell death activated by YTX after 48 h incubation, when the complex was located in the nuclear domain, was described as autophagy [67]. This programmed cell death activated by the toxin was also indicated in human glioma cells as a consequence of endoplasmic reticulum-stress, cell cycle arrest in G1 and inhibition of protein synthesis [68]. This last effect was also observed in myoblast cells, and YTX was proposed as a ribotoxin [69]. In K-562 cells, the YTX-activated autophagy was dependent of PDE4A but independent of PKC [58,67]. In addition to apoptosis and autophagy, paraptosis, a cytoplasm caspase-independent death mechanism, was also described after YTX and 1-desulfoYTX incubations [70,71]. This process was described in BC3H1 myoblast cell lines, as independent of caspases, and causing extensive cytoplasmic vacuolation, changes (swelling) in mitochondria and endoplasmic reticulum (ER), no DNA fragmentation and activation of mitogen-activated protein kinases (MAP kinase). Considering all these data about death activation, YTX is one of the few compounds reported to induce cell death by all three mechanisms, apoptosis, paraptosis and autophagy (Figure 3). Thus, as it was proposed, this compound is a valuable tool to study multiple death pathways [72].

**Figure 4:**
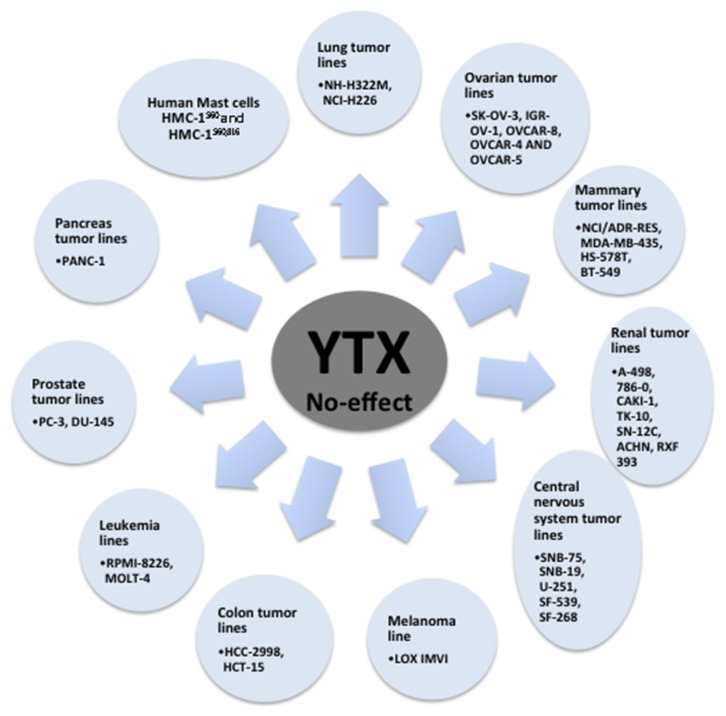
Summary of cellular lines no affected by YTX treatment [73].

The potential of YTX to induce cell death was checked in the 58 cell lines of the National Cancer Institute human tumor cell line screen [73]. From these results, high toxicity of YTX, in the nanomolar range, was observed on 27 of 58 cellular lines checked after 48 h incubation. High YTX sensitivity was observed in tumor lines from melanoma, seven affected of eight tested; lung, seven of nine; colon, five of seven; and leukemia, three of five. Medium sensitivity was reported in mammary tumor lines, three of seven. Low sensitivity in tumor lines from ovarian, one of six; renal, one of eight; central nervous system, one of six; and prostate, zero of two was also reported [73]. Reduction of cell viability was also reported in primary rat cardiomyocytes after YTX exposure [40]. However, no acute effects on cellular viability had been observed in non-tumor cells such as primary cerebellar mouse neurons, fresh human lymphocytes and fresh rabbit enterocytes after treatment with concentrations of YTX highly toxic for other cells [35,45,74]. On the contrary, high toxic effects were reported in fresh cortical mice neurons and in long cultured rat cerebellar neurons [39,56]. The effect in cell viability was further studied in a lymphoblastoid cell line. This is a non-tumor model with normal apoptotic and mitotic machinery [65]. Again, opposite to tumor models, no cell death activation was observed in these cells after 24 or 48 h in the presence of YTX. In this sense, variations in apoptosis hallmarks were not detected in the lymphoblastoid cell line after YTX incubation. On the contrary, some autophagy hallmarks were modified after 48 h of toxin incubation that converged into decrease in cellular proliferation, while no cell death was observed. Thus, YTX treatment triggered autophagy cell death in K-526 cells while in lymphoblastoid cells the toxin stops cellular proliferation. These YTX effects were related to PDE4A in both cellular lines. Therefore, the dual effect of YTX over tumor and non-tumor cells point to this compound as a powerful antitumoral drug or at least as a good drug lead [75,76]. In this sense, AKAP 149 protein is essential for mitosis [77,78,79], hence those factors that prevent the activity of this protein cause cell death, as in the case of YTX in K-526 cells. This line of reasoning based on AKAP 149 would explain the differences in response between normal and tumor cells to YTX. Indeed, surprisingly different molecular weights for PDE4A proteins were observed depending on the cellular model, 80 kDa in the case of K-562 cells, and 98 kDa, the normal molecular weight for this protein, in the lymphoblastoid cells [65]. In this sense, tumor cells have protein mutations that could lead to the change in the protein function, and these mutations are sometimes used as tumor biomarkers [80]. Therefore, YTX seems to be a specific cellular death inductor of tumor cells independently of their mitotic ability although the proliferation rate can be essential to explain YTX cellular resistance [68]. The variations of YTX effects over cell viability depending on the cellular model tested are shown in Table 1 (non-tumor cells), Table 2 and Figure 4 (tumor cells).

**Table 1:** YTX effects in non-tumor cells.

Cellular Model	Effect (YTX Concentration and Incubation Time)	Reference
Fresh enterocytes (rabbit)	No effect on F-actin (1 µM, 4 h)	[74]
Long cultured cerebellar neurons (rat)	Actin decrease, Apoptosis (5–150 nM, 48 h)	[39]
Primary cardiomyocytes (rat)	Irreversible reduction of cell viability (>10 nM, 48 h)	[40]
Primary cerebellar neurons (mouse)	No cellular death (1 µM, 48 h) (50 µM, 48 h, 70% cellular death)	[45]
MDCK kidney cells (dog)	Cellular Death Accumulation of E-cadherin fragment ECRA100 (1 nM, 21 h)	[62]
Fresh lymphocytes (human)	No effect on cell viability (1 µM, 48 h)	[35]
Lymphoblastoid cell line (human)	No effect on cell viability (30 nM, 24 h, no proliferation but no death)	[65]
Fresh cortical neurons (mouse)	Cellular death (1–100 nM, 48 h)	[56]

**Table 2:** Cellular lines showing different cellular death-types and effects induced by YTX.

Cellular Model	Effect	Reference
Rat glioma cells	Cell detachment and cytotoxicity	[33]
BE(2)-M17 human neuroblastoma cells	Apoptosis	[42]
HeLa S_3_ human cervix adenocarcinoma cells	Cellular death Apoptotic hallmarks	[59]
L6 and BC3H1, rat and mouse skeletal myoblasts	Cytoskeleton disruption Apoptosis	[43,60,81]
NIH3T3 mouse fibroblasts	Lysosomal damage, which may suggest autophagy	[61]
Bel7402 human hepatoma cells	Apoptosis	[36,38]
MCF-7 human breast adenocarcinoma cells	Cellular Death Accumulation of E-cadherin fragment ECRA100	[62,63]
A2780 human ovarian carcinoma and HeLa229 human cervix carcinoma cells	Cellular death	[45]
Hep G2 human hepatocellular cells	Apoptosis	[64]
BC3H1 myoblast cells	Paraptosis	[70]
Mouse T-lymphocytes EL-4 cells	Disruption of F-actin cytoskeleton Apoptosis	[82]
HL7702 human hepatoma cells	Apoptosis	[37]
Human Erythroleukemia K-562 cells	Apoptosis and autophagy	[55,67]
Human glioma cells	Autophagy	[68]
Mammary tumor lines MDA-MB-231, MCF-7, T-47D	Cellular death	[73]
Ovarian tumor lines OVCAR-3	Cellular death	[73]
Lung tumor lines A-549, HOP-92, EKVX, HOP-62, NCI-H23, NCI-H522, NCI-H460, MSTO-211H	Cellular death	[73]
Renal tumor lines UO-31	Cellular death	[73]
Central nervous system tumor lines SF-295	Cellular death	[73]
Melanoma line MALME-3M, SK-MEL-28, SK-MEL-2, SK-MEL-5, UACC-257, UACC-62, M-14	Cellular death	[73]
Colon tumor lines KM-12, COLO-205, HT-29, SW-620, HCT-116	Cellular death	[73]
Leukemia lines K-562, SR, CCRF-CEM	Cellular death	[73]
Pancreas tumor lines BxPC-3	Cellular death	[73]

## 6. Cytoskeleton and Cell Adhesion and YTX

After YTX incubation, cells detachment from culture dishes was observed [33,42]. However, no modifications in F-actin were reported, both in neuroblastoma BE(2)-M17 cells and rabbit fresh enterocytes, after 1 h or 4 h of 1 µM YTX exposure [74,83]. On the contrary, lysosomal vesicles, and progressive depolymerization of actin microfilaments were described as events activated after YTX exposure of insect fat body IPLB-LdFB cells, mouse fibroblasts NIH3T3 cell lines and cultured rat cerebellar neurons [12,39,61]. In addition, disassemble of F-actin and translocation of tensin during cytoskeleton disruption was described after 72 h YTX treatment when apoptosis was activated in L6 and BC3H1 myoblast cell lines [81]. Disruption of F-actin cytoskeleton was also observed in mouse T-lymphocytes EL-4 cells after 48 h of incubation the presence of YTX associated to apoptosis activation [82]. However, as it was mentioned only ultrastructural cardiac damages without other cellular alterations were reported after *in vivo* administration of YTX to rats and mice [6,7,8].

E-cadherin is a large family of proteins responsible for calcium-dependent cell-to-cell adhesion. This protein mediates in the aggregation-dependent cell survival. A decrease in E-cadherin expression is usually associated with the tumor expansion in epithelial cells, but also the protein has a role in survival and apoptosis suppression in other carcinoma cells [84]. E-cadherin collapse, with accumulation of a 100 kDa fragment of E-cadherin, without a parallel loss of the intact protein, was described after YTX incubation of human breast cancer cells MCF-7 and Caco-2 cells [85,86]. This effect was structure-selective and dependent on C9 chain [87]. In this sense, the same structure-selectivity relationship was observed in the association of PDEs and YTX analogs [49]. However, no *in vivo* effects over E-cadherin disruption were observed after mouse oral administration and E-cadherin stabilization in mouse colon cells was observed [88]. These contradictory *in vitro* and *in vivo* effects were later explained as an effect of YTX on E-cadherin degradation pathway, which is affected by the cellular context [62]. Therefore, again, different results were reported after YTX exposure depending on the cellular model.

## 7. Immune System and YTX

YTX has been reported to have an immune-regulatory effect on T-lymphocyte EL-4 cells by reversible down-regulation of the T cell receptor complex due to activation of [57,82]. On the other hand, as happens with drugs that decrease the levels of cAMP, YTX had increased the release of IL-2 in fresh human T cells [44]. In this sense, the release of cytokines such as TNF-α, was also activated by YTX in the murine macrophage cell line J774, and the toxin reduced the phagocytic activity and phagosome maturation of mouse peritoneal macrophages [89]. On the other hand, an increase in mussel phagocytic immunocytes was described under control conditions after YTX addition, but no effect was reported under stress conditions [12,90]. Indeed, shape changes in mussel immunocytes were detected after toxin exposure and although these cells were no activated, an increase in the active response of other activators was reported. In this effect, both extracellular calcium and cAMP were involved [12,91]. After mice intraperitoneal injection, structural damages in the thymus and variations in immune cells have been described [12]. Therefore either in mammalian or shellfish, YTX has some relation with the immune system and/or the immune response. In this sense, cAMP/PKA/PDEs pathway has a key role in cellular activation of inflammatory cells such as mast cells [92]. In this sense, YTX effect was also checked in fresh rat mast cells and in the Human Mast Cell lines HMC-1^560^ and HMC-1^560,816^. Mast cells are non-excitable mononucleated cells, mainly involved in episodes of inflammation and immune response. These cells are well known models frequently used in studies of immune response. The activation of mast cells induced after immunological stimulation was highly inhibited in the presence of YTX [93]. Besides in HMC-1 cell lines, inhibition of cellular stimulation and no modifications in cellular viability after 48 h incubation in the presence of YTX (1–100 nM) were observed (unpublished results). All these data point to YTX as an interesting tool in allergy processes [93].

## 8. Alzheimer’s Disease and YTX

An interesting pharmacological effect over Alzheimer’s cellular pathology had been described after YTX treatment. As was mentioned, YTX in the *in vitro* model of Alzheimer’s disease, primary cortical neurons 3xTg-AD, showed an improvement of Tau and β-amyloid levels through a mechanism related to the activation and translocation to the plasma membrane of cytosolic PKC. In this sense, it is known that patients with Alzheimer’s disease have reduced PKC levels [94]. Again, different response was observed depending on the cellular model. The response to YTX in primary transgenic neurons with three mutations and primary normal neurons was different regarding to PDE4. YTX increased 65% PDE4 levels in normal neurons, and no modifications in PDE4 levels were observed in transgenic neurons. In addition, in neurons, cAMP levels were not modified in the presence of YTX [56]. Moreover, from all these data, the effect of YTX in Alzheimer’s neurons is closer to therapy than to toxicology [95].

## 9. Glucose Metabolism and YTX

Important transcriptional changes on the lipids and glucose metabolism have been described after YTX treatment of glioma cells [68,73]. Alterations in pancreas and liver, with fatty degeneration were reported earlier as consequence of di-desulfo-YTX exposure [15]. A deregulation of lipid metabolism induced by the toxins was observed in glioma cells as consequence of endoplasmic reticulum stress. This effect translates into an increase in cholesterol content. This increase may justify the increase in cytosolic calcium produced by the toxin. The stress induced is also related to an increase in mitochondrial metabolism [68]. From these experiments, YTX was proposed as a lead molecule to treat and/or prevent metabolic diseases [96].

## 10. Conclusions

**Figure 5:**
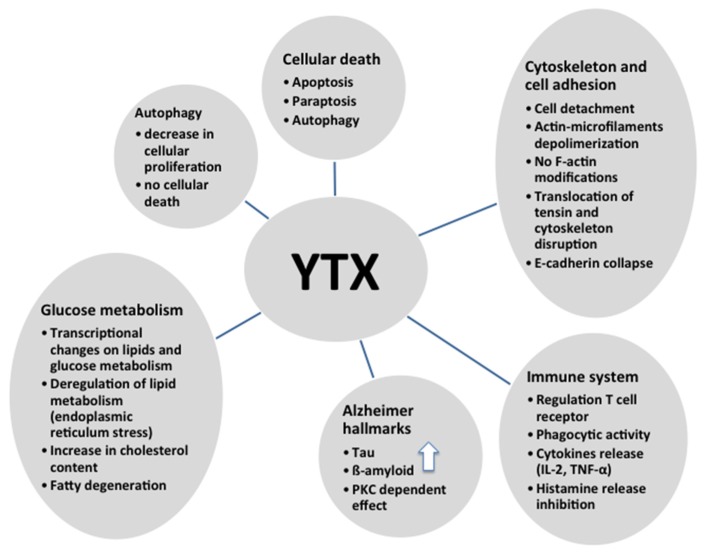
Summary of different effects of YTX.

As shown in this review, YTX has multiple effects and therefore is a powerful tool to study different intracellular pathways (Figure 5). Since different, and sometimes contradictory, effects were observed depending on the cellular model, it is very difficult to confirm that this compound is only a toxin. However, it is a helpful drug for pharmacological approaches, besides some of the YTX effects are potentially useful with therapeutic proposals.
